# Redefining Obesity: Implications for Holistic Nursing Practice

**DOI:** 10.7759/cureus.78134

**Published:** 2025-01-28

**Authors:** Abdulqadir J Nashwan

**Affiliations:** 1 Nursing & Midwifery Research Department, Hamad Medical Corporation, Doha, QAT

**Keywords:** body mass index (bmi), nursing education, obesity, patient-centered care, weight stigma

## Abstract

The Lancet Diabetes & Endocrinology Commission's recent redefinition of obesity as a chronic, systemic illness marks a paradigm shift with profound implications for nursing practice. Moving beyond body mass index, the framework emphasizes holistic assessments, integrating validated adiposity and organ dysfunction measures. Nurses are uniquely positioned to address weight stigma, advocate for equitable care, and implement evidence-based interventions. They can provide empathetic, non-judgmental care, create inclusive environments with appropriate resources, and use motivational interviewing to support patients’ health goals without overemphasizing weight loss. Additionally, advocating for anti-bias training and equitable care policies ensures a stigma-free, patient-centered approach. This editorial highlights how the redefinition of obesity necessitates enhancements in nursing education and policy advocacy, equipping professionals to manage obesity's multifaceted challenges effectively.

## Editorial

The Lancet Diabetes & Endocrinology Commission’s recently published framework on the definition and diagnostic criteria of obesity presents a critical evolution in understanding obesity as a systemic, chronic illness characterized by organ dysfunction due to excess adiposity [1]. This reconceptualization has profound implications for nursing practice, particularly in fostering patient-centered care and addressing systemic biases within healthcare. Key practices include engaging patients in shared decision-making, respecting their values and preferences, and delivering individualized care that considers their unique needs. Nurses can also actively identify and challenge systemic biases by promoting equitable access to resources and creating inclusive environments that support diversity. These practices enhance patient satisfaction and outcomes and contribute to building trust and fostering meaningful therapeutic relationships.

Obesity is a global health crisis, affecting over 650 million adults and 340 million children and adolescents worldwide, as estimated by the World Health Organization (WHO) [2]. The prevalence of obesity has tripled since 1975, with more than 39% of adults overweight and 13% classified as obese [2]. The condition contributes significantly to non-communicable diseases, including type 2 diabetes, cardiovascular diseases, and certain cancers, accounting for approximately 2.8 million deaths annually [2]. Economically, obesity-related healthcare costs are projected to surpass $1 trillion globally by 2025 [2]. This epidemic underscores the urgent need for comprehensive prevention and management strategies across healthcare systems and communities. However, several obstacles must be addressed to ensure the success of these strategies. Economic limitations often hinder the availability of resources and access to care [2]. Time constraints, particularly for healthcare providers balancing multiple responsibilities, can limit the implementation of preventive measures and personalized care plans. Provider resistance to adopting new practices or guidelines, often due to lack of training or skepticism, presents another challenge. Additionally, the digital gap exacerbates disparities, as individuals in underserved or rural areas may lack access to technology-based interventions, such as telemedicine or health apps [2]. Overcoming these barriers requires a multifaceted approach, integrating policy changes, education, and investment in infrastructure to support equitable and practical solutions.

Obesity has long been measured and understood predominantly through body mass index (BMI), a metric criticized for its limitations in assessing individual health [3]. The Commission rightly challenges this paradigm by emphasizing the need to confirm excess adiposity using validated anthropometric or direct fat measurement methods and to diagnose clinical obesity based on organ or tissue dysfunction or significant impairments in daily living [1]. Such refined criteria enable more accurate clinical assessments and interventions tailored to individual patient needs. Direct fat measurement techniques provide more precise body composition evaluations, including dual-energy X-ray absorptiometry (DXA) and bioelectrical impedance analysis (BIA). Functional assessments, such as physical performance tests and evaluation of organ dysfunction (e.g., liver fibrosis scans for non-alcoholic fatty liver disease), are crucial for diagnosing clinical obesity [1].

This redefinition of obesity underscores the importance of holistic health assessments, a cornerstone of nursing practice. Nurses are pivotal in patient care, particularly health promotion and disease prevention, especially obesity [4]. The expanded criteria for diagnosing obesity encourage nurses to adopt a more comprehensive approach in evaluating patients, moving beyond reliance on BMI to include assessments of waist circumference, functional limitations, and other validated measures. Such an approach ensures a more accurate depiction of a patient's health status, allowing for tailored care planning that aligns with the specific needs of the individual.

The framework also addresses the pervasive issue of weight-based bias and stigma in healthcare. Such biases not only exacerbate health disparities but also hinder effective treatment. The nursing profession, often serving as the frontline of patient interaction, is uniquely positioned to combat these stigmas. Nurses' advocacy for equitable policies that ensure access to care further reinforces this commitment to justice and inclusivity.

Another significant implication of the redefined criteria for obesity is the integration of evidence-based interventions into nursing practice. Nurses can harness this framework to guide their interventions through lifestyle counseling, pharmacological support, or referrals for surgical interventions when necessary. Evidence-based approaches allow nurses to focus on improving clinical outcomes for patients with obesity, helping them manage complications and enhance their quality of life.

Advocacy for policy changes is a vital aspect of nursing practice that gains renewed importance with this redefinition of obesity [5]. Nurses can influence public health strategies by emphasizing the importance of early detection and intervention for preclinical obesity. For example, public health nurses in community settings can spearhead early detection programs through school-based screenings, identifying children at risk for obesity and initiating preventive interventions. Similarly, hospital-based nurses can integrate evidence-based protocols into discharge planning, ensuring that patients receive appropriate referrals to nutritionists or weight management clinics. The distinction between preclinical and clinical obesity highlights the necessity of preventive measures and efficient resource allocation. As advocates, nurses can contribute significantly to shaping policies that address these priorities.

Redefining obesity also necessitates enhancements in nursing education and training. Preparing nurses to address the complexities of obesity involves incorporating training on advanced diagnostic tools, culturally sensitive care approaches, and the management of obesity-related complications into curricula [6]. Cultural competence is vital in addressing diverse dietary practices, body image perceptions, and barriers such as stigma and language differences [6]. It enables nurses to build trust, tailor interventions to patients’ cultural contexts, and improve adherence and outcomes [6]. Incorporating this training ensures nurses are prepared to deliver effective, patient-centered care for individuals with obesity. Such education equips nurses to provide comprehensive and effective care to patients with obesity, ensuring they are well-prepared to meet the challenges posed by this chronic condition.

The practical implications of this redefined framework extend to several key aspects of nursing practice, including screening, care planning, and community health initiatives. Screening protocols in healthcare settings may evolve to incorporate anthropometric measurements, such as waist circumference, and functional assessments, such as physical performance tests, as routine evaluations. Care planning must emphasize developing multidisciplinary pathways that address both the medical and psychosocial dimensions of obesity, integrating nutrition, mental health support, and lifestyle counseling.

This framework becomes even more relevant when considering geographic and healthcare system variations. For example, in low-resource settings, cost-effective and easily implementable screening tools may be prioritized, while advanced diagnostic technologies could be leveraged in high-resource settings. Community health nurse-led initiatives, such as culturally tailored education campaigns or mobile clinics, can address preclinical obesity and promote healthier lifestyles, especially in underserved or rural areas. Adapting these strategies to local contexts ensures they are effective and equitable, addressing the unique challenges of diverse populations and healthcare environments.

To sum up, redefining obesity as a systemic chronic illness offers an opportunity to transform nursing practice by fostering a holistic, equitable, and evidence-based approach to care. Nurses, as integral members of the healthcare team, can play a pivotal role in mitigating the burden of obesity on individuals and healthcare systems by addressing its multifaceted impacts.
